# Practical Remarks on the Diseases Which Occurred on Board the Astrea, &c.

**Published:** 1799-06

**Authors:** Stewart Henderson

**Affiliations:** No. 7, Lancaster Court, Strand


### *Practical Remarks on the Diſeaſes which occurred on board the Aſtrea, &c. By* STEWART HENDERSON.

[*Concluded from our laſt Number,* pp. 236—238.]

### EPIDEMIC CATARRH.

THIS catarrh, reſembling the influenza, which was ſo prevalent in England and other parts of Europe in 1782, made its appearance on board the Aſtrea the 5th of October, 1789, when at anchor off Kingſton: in the courſe of a few days there was hardly a man in the ſhip but was attacked in a greater or leſs degree; moſt of the ſquadron felt its influence; but it was obſerved to ſeize moſtly thoſe who had been longeſt on the ſtation. The Centurion and Blonde, ſhips newly arrived from England, had hardly any taken ill. The people in the town of Kingſton, eſpecially the lower claſs of whites and negroes, who ſleep in out-houſes, were generally affected. The appearance of the diſeaſe at different periods pervaded the whole iſland, ſometimes prevailing in one place a week before it was felt in another; as happened to the 10th regiment, ſtationed at Fort Auguſta, which is only four or five miles from where the ſquadron lay.

The ſymptoms varied in different perſons; in ſome they were complicated with the endemic remittent; in general they were ſeized ſuddenly with chillineſs chillinefs and fhivering, fucceeded by a hot fit, langour, and laffitude; pain and ftricture acrofs the thorax, with fome difficulty of breathing; anxiety, ficknefs, and violent pain in the head, particularly above the orbits; frequent fneezing, the membrana fchneideriana much affected; heat and forenefs in the throat, with frequent cough. Some complained of a violent pain in the loins, which was much increafed upon motion; one had a bleeding from the nofe; delirium appeared in fome, but only in thofe who had catarrhal fymptoms, complicated with the remittent fever; the tongue white, and in fome yellow; the pulfe quick and fmall; the fkin dry; the belly, in general, coftive. Though the pain of the breaft and fide was fometimes violent, yet the pulfe was feldom hard; no mark of inflammatory diathefis fo as to indicate bleeding; debility commenced with the difeafe and continued after the catarrhal fymptoms were gone, requiring the ufe of bark and the moft powerful cordials; relapfes were frequent, from being expofed to the weather, but the fymptoms were in a milder degree.

This difeafe appeared to me to be occafioned by the fudden tranfition of the weather. The preceding month had been uncommonly hot, fuch as had never been felt by the oldeft inhabitant in Kingfton; the thermometer in the day, being never below 90 in the fhade. During this month, people were afraid to ftir out; feveral from being expofed to the intenfe rays of the fun were feized with phrenzy, which in two inftances, I was told, proved fatal. The fea-breezes were pretty regular; but we had not the ufual rains and fqualls which diftinguifh this feafon, and characterize what are called the hurricane months; partial fhowers, indeed, fell in the mountains, but did not reach the vallies. In the firft week of November the thermometer fell 10 degrees in the day, and experienced a much greater change in the night: the fea breezes left us; the air became fo foggy as to obfcure the folar rays; a degree of dampnefs was felt, and in the night, a ftrong land wind blew from the mountains, carrying with it a fenfible degree of moifture. Every one who was expofed to this air was fuddenly attacked with the difeafe. All thofe men who flept upon deck without any covering, which many were induced to do from the great heat below, fuffered from their imprudence; for they had the complaint in a more violent degree than the reft who were afterwards feized.

I remarked, that the men who flept below in the cable-tier were the laft feized, and had the complaint in a flighter degree; but the fymptoms not being fo violent, might be owing to the pre-difpofing caufe being fomewhat abated, on a change of the weather. For about the latter end of the month, the fea-breezes returned, with fqualls, which foon difperfed the furrounding vapours, and gave a brifk circulation to the ftagnated air. With this change the difeafe difappeared.

The Centurion and Blonde not being affected, proved clearly that no contagion existed in the air. I imagine their exemption from it was owing to the constitutions of the men not having arrived at that degree of relaxation and irritability, which a continuance of some years in a hot climate never fails to produce, and renders the habit more susceptible of the sudden influence of cold, which appears to have been the sole cause of this epidemic catarrh.

In the beginning of the complaint, an emetic was generally given, which relieved the nausea and sickness, and produced some degree of perspiration. This was encouraged by keeping the patients in bed, and causing them to drink plentifully of warm sage-tea, barley-water, &c. From the number of men confined below, as well as the nature of the epidemic, and state of the air, I thought it necessary to keep buckets of hot vinegar under their hammocks, to correct the stagnated air. Antimonials, with the tinct. opii, were administered; the vin. antimon. was what I in general used, thirty to forty drops four times a day, adding to each dose ten drops of the opium tincture; and by the assistance of the warm bath a plentiful perspiration was kept up. To relieve the cough, and promote expectoration, the sp. minder, with the oxymel scillet. were ordered. The tart. emetic, given so as not to produce full vomiting, afforded great relief to the febrile symptoms, by promoting perspiration, and removing the mucus accumulated in the bronchiæ. When the pain and stricture in the thorax were violent, blisters gave relief. Bark was given in those cases which partook of the remittent type; and, on account of the debility which commenced and continued with the disease, wine was often necessary. From the same cause, bleeding was never made use of on board the Astrea. Indeed, I found that the men from being so long exposed to the debilitating effects of a hot climate, bore evacuations very ill. These were the principal means we employed for the cure of this catarrh, and when unattended with pulmonary symptoms, it soon yielded to them. In those cases which terminated by expectoration, the debility was very great, requiring a nourishing diet, and plenty of good wine, which was never spared on these occasions.

ULCERS.

Of ulcers, and the nature of the ulcerative process, much has been said of late by eminent medical characters. It is only my intention here to take notice of that ulcer to which seamen and soldiers are more particularly subject, from the nature and manner of their life. Next to fever and dysentery, this complaint proves the most destructive to British seamen and soldiers, and no surgical disorder has deprived his majesty's service of so many men, as every experienced surgeon must have observed, who has attended our hospitals during during war; nor are there any cases more unfit for general hospitals on their present principle.

When I acted as surgeon to his majesty's naval hospital at Antigua, in 1780, nearly one third of the patients laboured under this complaint.—Being at that time a young surgeon, I was unwilling to run the risk of deviating from established authorities, and therefore pursued the plan pointed out by men of high repute in the profession. Our method of treatment then consisted of poultices, various ointments, and other relaxing applications, with a sparing use of tonics and stimulants; rest and confinement were strictly enjoined. I could not help lamenting the inefficacy of those remedies, when I saw so many fall victims to the disease, and others lose their limbs, by being obliged to undergo a painful and hazardous operation. Baffled in every attempt to heal these ulcers, I was under the necessity of having recourse to an operation, as the only chance of saving life.

Dr. Young, physician to the hospital, Mr. Weir, surgeon of the Alcmena, now physician to Lord St. Vincent's fleet, agreed with me in the necessity and propriety of performing the operation. We considered that it was only assisting nature, for she was endeavouring to get rid of a part which had become useless, and was contaminating the whole system. I was further encouraged by the anxiety of the patients to have it done; and what gave me greater reason to expect success, was, that in a row of empty rooms appropriated for officers, I had the liberty of putting the men before the operation, where they would breathe pure wholesome air, which they had long been strangers to in the hospital. Fourteen underwent the operation, most of them below the knee, which I always prefer when the case will admit of it. Such was the advantage of their being removed from the noxious air of a crouded ward, that in a few days all their hectic symptoms left them; appetite returned; the stumps put on a very healthy appearance, and continued to do so, until the ulcer contracted to about the size of a half-crown, when it seemed to be stationary, discharging a thin ichor. Though their appetites were good, and notwithstanding they had a nourishing diet, and good Madeira, and at the same time bark and other tonics, they did not appear to gain strength. From their pale and languid countenances, I judged that moderate exercise in the open air, in the morning and evening, with the use of the cold bath, which happened to be convenient, might have a good effect in restoring their general health. I immediately procured crutches for them, and had them plunged into the salt water every morning. The good effect was soon visible, not only in their countenances, but the sore put on a fine, florid, and healthy look, discharging good pus, and contracted daily. They all recovered, except one man, a landsman, who was a very irregular liver, and who, notwithstanding withſtanding that every precaution was taken to prevent it, procured and drank immoderately of new rum; part of a bottle of this poiſonous liquor being found in his bed when he died.

The bad effect of foul air on ulcers I had an opportunity of witneſſing at the Cape of Good Hope. A great number of the ſeamen and ſoldiers had ulcers, occaſioned by the immoderate uſe of ardent ſpirits, and their not uſing ſufficient vegetable aliment. The climate itſelf is remarked for its ſalubrity, and the Britiſh army for a conſiderable time paſt have been in a ſtate of health, unknown in any other part of the world. Storehouſes appropriated for the reception of the ſick, were then extremely crouded, and the air ſo vitiated, that the ſlighteſt caſe degenerated into the moſt malignant, in the courſe of a few days. Notwithſtanding every effort of very able ſurgeons, numbers died, and many loſt their limbs; none I believe recovered from the operation, until they thought of removing them to ſeparate apartments, which had the ſame good effect as at Antigua.

From what I have obſerved of the bad effect of crouding patients with ulcers, fever, or dyſentery, it appears, that the beſt and moſt powerful remedies will fail, when the patients are deprived of that great pabulum of life, pure atmoſpheric air, on which the preſervation and reſtoration of health ſo much depend. Three feet, the greateſt ſpace generally allowed between the beds, is not ſufficient, in wards containing acute infectious diſeaſes and ulcers; the fœtor ariſing from one large ulcer, will contaminate a whole ward, and render the air noxious to animal life.

On finding ſuch beneficial effects from exerciſe, pure air, and cold bathing, in accelerating the healing of the ſtumps, I was led to try what theſe combined would effect in the cure of the ulcers.

The ulcers to which ſeamen and ſoldiers are particularly ſubject, are brought on, as I have before remarked, from their not uſing ſufficient vegetable aliment, joined to irregularities, eſpecially the abuſe of ſpirits, heat and moiſture, and expoſure to impure air. In a conſtitution previouſly prepared by ſuch debilitating powers, we can eaſily conceive, that the ſmalleſt ſcratch will degenerate into an ulcer, which, when below the knee, becomes very difficult of cure, often baffling every attempt of the healing art. This ariſes from the ſtate of the fluids, and general debility, but particularly in the extreme parts, where there is a greater deficiency of vital principle, even in health. Perhaps the tendinous ſtructure of the parts, and their depending ſituation, may aſſiſt in retarding the cure of ulcers ſituated upon the lower extremities.

Appearance

*Appearance of the Ulcer.*

The ulcer generally makes its appearance on the fore or inner part of the leg, near the ancle, and discharges a thin acrimonious matter, excoriating the surrounding parts. On neglect or improper treatment, it enlarges daily; the discharge becoming more and more acrid, attended with a putrid fœtor; fungous excrescences begin to rise; the limb becomes œdematous, and very painful. Sometimes, on the least touch, or depending position, a hæmorrhage takes place; the bones become carious; putrid sloughs are cast off daily; and, from the constant irritation, the patient is kept in continual pain; has restless nights; and a hectic fever with colliquative sweats succeed. In this state, if the operation be not soon performed, a diarrhœa generally comes on, which in a few days closes the scene.

The progress of these symptoms will depend more or less on the constitution of the patient, the putrid diathesis in the system, exposure to miasmata, and in general to all the predisposing causes. In our expedition to St. Juan's, during the last war, such was the state of the air at that place, as I was informed by a surgeon in actual service there, that the slightest wound on the lower extremities degenerated into the worst symptoms I have described of this ulcer, in the course of three days, and carried off the patient in spite of the most powerful antiseptics.

*Cure of the Ulcer.*

In the cure of the ulcer, I have found that all relaxing and emollient applications are improper, unless when pain and inflammation require their use: when these symptoms are removed, they should be laid aside. The external applications, which in a great number of cases have succeeded with me, were warm and stimulant, increasing the action of the extreme vessels, and restoring the parts to their proper tone. With this view, I used the hydrargyr. nitrat. rubr. in powder, and sometimes in the form of ointment, or the solution of arg. nitrat. I often found it necessary to vary those escharotics: or leave them off when the discharge was copious, and apply dry lint, with slips of cerate to defend the edges; and over that a compress of linen, wet in a solution of sacchar. saturn, with the addition of a little camphorated spirit, supported by a roller of thin flannel or cotton, which was moistened in camphorated vinegar, renewed two or three times a day, and continued from the extremity of the foot to a little below the knee, not to prevent the motion of the joint, nor the action of the muscles. This enabled the patient to use moderate exercise, which I strictly ordered as soon as the pain and inflammation abated. This, with the daily use of the cold salt-water bath, greatly accelerated the healing process, and assisted aſſiſted in reſtoring the general health. When the diſcharge was fœtid, lint dipped in tincture of myrrh, bark, or carrot poultices, corrected the fœtor, and lime-juice proved a good detergent..

While theſe external means were employed, internal remedies were at the ſame time adminiſtered, with two intentions; firſt, to correct the putrid diatheſis and produce good juices; ſecondly, to brace and ſtrengthen the ſyſtem.

To anſwer the firſt, the bark was given in copious doſes; lime-juice, and a large proportion of vegetable aliment, with plenty of good wine; while evacuations, which might leſſen the vital principle, as well as debilitating cauſes, were carefully avoided. The ſecond was effected by preparations of ſteel, aſſiſted by exerciſe, and the cold bath: but what I conceived to be the moſt efficacious, was a very liberal allowance of pure atmoſpheric air, which I conſidered to be of greater importance than all the other remedies; for, deprived of that, all the reſt would effect no ſalutary purpoſe. The incalculable miſchief which the want of that ſovereign remedy has produced in our hoſpitals is well known, and experienced by every medical man of obſervation.

In concluding theſe few remarks, I am ſenſible that the ſucceſs we met with did not depend on any particular mode of treatment, differing from the common practice; but was more owing to having it in our power to attack diſeaſe on its firſt commencement, before morbid movements had made much impreſſion; thereby affording us an opportunity of cutting ſhort the diſeaſe, or at leaſt obviating its danger. At the ſame time, cleanlineſs and wholeſome air were particularly attended to, and every cauſe removed which was likely to aggravate ſymptoms, or counteract the effect of medicine; while Capt. Rainier very humanely allowed the ſick wine and freſh diet from his table. Without thoſe aids, all our medicines, I fear, would have availed but little.

*No. 7, Lancaſter-Court, Strand,*

*March 16th, 1799.*

Stewart Henderson.

A TABLE

